# Deep Eutectic Solvent-Assisted Synergistic Technologies for the Extraction of Natural Bioactive Compounds: Enhancement Mechanisms, Application Advances, and Challenges

**DOI:** 10.3390/foods15152611

**Published:** 2026-07-25

**Authors:** Wenrong Meng, Wenhao Hu, Tiancheng Sheng, Qiang Chen, Fei Lu, Longkun Wu

**Affiliations:** 1 School of Food Science and Technology, Shenyang Normal University, Shenyang 110031, China; 15524498151@163.com (W.M.); huwh0306@163.com (W.H.); 19359918041@163.com (T.S.); 2 College of Life Sciences, Shenyang Normal University, Shenyang 110031, China; wukuner2@163.com

**Keywords:** mass transfer, viscosity, hydrogen bonding, polyphenols, natural deep eutectic solvents (NADES), process intensification

## Abstract

Deep eutectic solvents (DESs) have emerged as promising green extraction media for natural bioactive compounds, offering tunable physicochemical properties, biocompatibility, and lower toxicity than conventional organic solvents. However, the high viscosity of DESs limits mass transfer efficiency, necessitating integration with auxiliary technologies to overcome this intrinsic constraint. Unlike previous reviews that focus on DESs coupled with a single auxiliary technique, this review for the first time integrates DESs with ultrasound, microwave, enzymatic assistance, and multi-technique hybrid systems into a unified framework, critically comparing their enhancement mechanisms, applicability boundaries, and industrial feasibility. This review systematically examines the non-covalent interactions between DES components and target compounds, including hydrogen bonding, van der Waals forces, and electrostatic interactions, and discusses how these interactions guide the rational selection of hydrogen bond acceptors (HBAs) and donors (HBDs) for specific compound classes. We analyze the distinct mechanisms by which ultrasound, microwave, and enzymatic assistance synergistically enhance DES extraction performance, with particular attention to viscosity reduction, cell wall disruption, and biocatalytic compatibility. Recent advances in the extraction of polyphenols, alkaloids, terpenoids, and polysaccharides are summarized to illustrate the practical application of these principles. Finally, we discuss current challenges in industrial scale-up, including solvent recovery, equipment adaptation for high-viscosity media, and regulatory considerations, and outline future directions toward intelligent solvent design and continuous processing. By integrating molecular-level mechanistic insights with process engineering perspectives, this review provides a comprehensive framework for the optimization and application of DES-based extraction technologies.

## 1. Introduction

Over the past few years, the rapid expansion of the functional food, natural medicine, and green cosmetics markets has driven sustained growth in demand for natural bioactive compounds such as polyphenols, flavonoids, saponins, polysaccharides, and alkaloids. These compounds are highly valued in the food and pharmaceutical industries owing to their pronounced biological activities, including antioxidant, anti-inflammatory, hypoglycemic, and immunoregulatory properties [1]. Efficient extraction of natural bioactive compounds is essential for realizing their medicinal and economic value. However, conventional extraction methods typically rely on volatile organic solvents such as methanol and acetone, which suffer from several drawbacks: their high toxicity and potential residues compromise product quality; high extraction temperatures readily degrade heat-sensitive compounds; and high energy consumption and large waste volumes are incompatible with green production principles. Moreover, regulatory frameworks such as the ICH Q3C guideline [2] and EU Directive 2009/32/EC [3] impose strict limits on residual solvents—Class 1 solvents (e.g., benzene, carbon tetrachloride) are prohibited, and Class 2 solvents (e.g., methanol, acetonitrile) are restricted by permitted daily exposure limits—while the limited selectivity of conventional solvents complicates downstream purification [4]. These methods are therefore increasingly at odds with the principles of green chemistry and sustainable development [4], and efficient, environmentally friendly alternative extraction technologies are urgently needed.

The term “deep eutectic solvents” (DESs) was first coined by Abbott and co-workers in 2003. Owing to their similarity to ionic liquids (ILs), DESs were initially regarded as a new class of ILs; however, they offer a far wider compositional range. Formed as eutectic mixtures through hydrogen bonding between hydrogen bond acceptors (HBAs) and hydrogen bond donors (HBDs), DESs exhibit melting points far lower than those of their individual constituents. Their tunability, low toxicity, high biocompatibility, and ease of preparation make them attractive alternatives to conventional organic solvents [5]. However, the “green” credentials of DESs should not be generalized. The toxicity of DESs varies substantially with the nature of the HBA and HBD, their molar ratio, and concentration. Food-grade DESs predominantly employ choline chloride or NADES with organic acids, polyols, or sugars as donors, which generally exhibit low cytotoxicity. Nevertheless, even these formulations can induce cytotoxicity exceeding that of their individual components under certain conditions. Metal-containing DESs (e.g., ZnCl_2_-based) exhibit notably higher toxicity and should be excluded from the processing of edible products. These findings indicate that DESs should be regarded as new chemical entities with distinct toxicological profiles, rather than as inherently benign merely because of their natural-origin constituents.

The mass transfer constraints that are imposed on natural product extraction like the inability to rupture cell walls and slow solute diffusion are fundamental bottlenecks limiting the efficiency of the process. Physical field (ultrasound, microwave, infrared) and enzyme-assisted technologies can have a very positive effect on the efficiency of extraction when used directionally to enhance mass transfer processes [6]. Ultrasonic-assisted extraction uses the microjets and shockwaves created through cavitation to destroy the cell wall structure of plants, thus allowing an increase in the surface area where solvent–solute contact occurs. As an example, DES–ultrasonic synergistic extraction of rosmarinic acid from calendula at 240 W ultrasonic power for 35 min achieved a 39.85% extraction yield, much higher than that of conventional solvent-based extraction [7]. Microwave-assisted extraction is the direct use of high-frequency electromagnetic fields to the material inside to burst cell structures that induce the release of constituents. Its fast heating characteristics coupled with low energy consumption and small extraction time allow its application to the fast extraction of heat sensitive compounds. The heating of the DES with microwaves minimizes its viscosity and improves the performance of the system on mass transfer. The high viscosity of DESs (typically >100 cP, with some glycosylated DESs reaching several thousand cP) stems from their dense hydrogen-bonding network. Although this dense hydrogen-bonding network endows DESs with excellent solubilization capacity, it simultaneously imposes kinetic constraints on the extraction process. According to the Stokes–Einstein equation, high viscosity reduces the diffusion coefficient of solute molecules, limiting their mass transfer from the plant matrix. Therefore, the extraction efficiency of DESs is fundamentally governed by a trade-off between solubility (thermodynamic advantage) and mass transfer rate (kinetic limitation), and the core value of synergistic technology lies in overcoming this bottleneck by reducing viscosity.

D = kBT/6πηr

where D is the diffusion coefficient of the solute, kB is the Boltzmann constant, T is the absolute temperature, η is the dynamic viscosity, and r is the hydrodynamic radius of the solute.

When used synergistically, these techniques increase the solubility and stability of target components; for example, synergistic DES–microwave extraction of polyphenols from orange peel at a microwave intensity of 664 W/cm^2^, a frequency of 20 kHz, and a duration of 1 min yielded an average polyphenol content of 4.12 ± 0.20 mg GAE/g with high antioxidant activity [8]. Enzyme-assisted techniques reduce mass transfer resistance by specifically hydrolyzing cell wall components (e.g., with cellulase and pectinase). For example, composite-enzyme (cellulase–pectinase)-assisted DES extraction of pectin from apple pomace increased the pectin extraction rate by 32% after 2 h of enzymatic hydrolysis and produced a more uniform pectin molecular-weight distribution [9]. Moreover, physical fields can be further combined with enzymatic methods to overcome mass transfer barriers: in the ultrasound–enzyme synergistic extraction of polysaccharides from kelp, ultrasonic cavitation enhanced enzyme–substrate interactions, quadrupled the enzymatic efficiency, and achieved a polysaccharide extraction yield of 25.6% [10]. By directionally intensifying mass transfer processes, these techniques help overcome efficiency bottlenecks in natural product extraction and provide important support for the industrial implementation of DESs.

Currently, research on deep eutectic solvent (DES)-assisted extraction technology is largely confined to the application and optimization of a single auxiliary technique. The core paradigm of these studies is the combination of DESs with a specific auxiliary method (such as ultrasonic-assisted extraction (UAE), microwave-assisted extraction (MAE), or enzyme-assisted extraction), wherein researchers optimize process parameters (such as solid-to-liquid ratio, temperature, time, and power) to improve the extraction yield of target compounds. For example, studies on agricultural food waste have focused on DES–ultrasonic-assisted extraction of polyphenols and other active components, thereby enhancing extraction efficiency through optimization of ultrasonic power (300–400 W), time (30 min), and temperature (40 °C) [11]. Further research has systematically investigated the application of DESs for extracting polyphenols from edible plants, thereby demonstrating their superiority as a green alternative solvent and elucidating how factors such as DES component concentration, pH, temperature, and water content influence the extraction process [12]. Additionally, comparative studies have evaluated different DES formulations and auxiliary techniques for extracting natural bioactive components, thereby confirming the feasibility of combining DESs with physical fields or enzyme technologies to improve efficiency and selectivity [13]. Several published reviews have also summarized DES-based extraction of natural bioactive compounds, either from a general solvent-design perspective [13,14] or for specific compound classes [15]; however, these reviews predominantly address DES coupled with a single auxiliary technique and do not provide a cross-technology comparison of synergistic mechanisms, applicability boundaries, or scale-up considerations within a unified framework. Overall, the aforementioned research generally adheres to the paradigm of combining DES with a single auxiliary technique (such as UAE, MAE, or enzyme assistance). This focus on a single technique makes it difficult to compare the efficiency of individual DES-assisted methods with that of synergistic multi-technique systems (such as ultrasound–enzyme, microwave–ultrasound, or ultrasound–-microwave–enzyme co-extraction). Because experience gained from optimizing a single technique is often difficult to apply directly to multi-technique scenarios, the extraction efficiency, selectivity, and versatility of DES-assisted extraction are severely limited when dealing with complex matrices containing multiple active components (such as polyphenols, alkaloids, terpenes, and polysaccharides simultaneously), which also restricts the scale-up from laboratory optimization to industrial-scale application.

Single extraction procedures are inherently limited: DESs offer strong solubilization capacity, but their high viscosity hinders mass transfer; physical field-assisted processes enhance mass transfer rates but lack selectivity toward target components; and enzyme-assisted systems selectively degrade cell walls, but their stability is susceptible to changes in the solvent environment. Synergistic enhancement aims to combine DESs with supplementary methods to achieve “1 + 1 > 2” effects. For example, in DES–ultrasound synergistic extraction, the solubilizing capacity of DESs couples with ultrasonic cavitation, simultaneously enhancing both target-component solubility and mass transfer. Compared with DESs alone, polyphenol extraction yields increased several-fold [16]. Under specific conditions, DES–UAE co-extraction can significantly improve extraction efficiency relative to single DES extraction; however, such enhancements are highly dependent on the raw material matrix, DES formulation, and process parameters, and should not be generalized. In DES–enzyme synergistic extraction, the mild nature of DESs preserves enzyme activity, while the selective enzymatic degradation of cell wall constituents facilitates DES penetration, increasing the alkaloid extraction rate by 30% [17].

This review systematically integrates the flexible design of DESs and auxiliary extraction techniques to overcome the limitations of traditional methods in mass transfer. Unlike the existing literature that focuses solely on a single coupling technique, this review aims to establish a comprehensive cognitive framework from molecular mechanisms to process applications, specifically exploring the following three core dimensions: (i) The interaction mechanisms between DESs and target bioactive compounds. The review thoroughly analyzes how non-covalent interactions such as hydrogen bonding, electrostatic interactions, van der Waals forces, and π–π stacking determine the solubility and selectivity of DESs for different classes of natural products, and on this basis, proposes rational design strategies for polyphenols, alkaloids, terpenes, and polysaccharides. This mechanistic discussion provides a theoretical foundation for the subsequent selection of synergistic techniques—for example, DESs with high hydrogen bond density are more suitable for ultrasonic assistance to overcome viscosity limitations, whereas DESs with high dielectric constants are better matched with microwave heating. (ii) The principles of synergistic effects when DESs are combined with ultrasound, microwave, and enzyme-assisted techniques. The review not only presents the independent mechanisms of each auxiliary technique (ultrasonic cavitation, volumetric heating by microwaves, enzymatic selective hydrolysis) but also focuses on analyzing the matching rules between DES characteristics and auxiliary techniques: ultrasound reduces mass transfer resistance in high-viscosity DESs by disrupting hydrogen bond networks; microwave rapidly lowers DES viscosity and promotes the release of target components through selective polarization heating; enzyme-assisted techniques maintain activity in the mild DES environment to achieve selective cell wall degradation. Additionally, the review critically examines multiple synergistic techniques, highlighting the current ambiguity in distinguishing between “synergistic effects” and “additive effects” in the literature, and emphasizes the necessity for quantitative assessment. (iii) The latest advances in the extraction of polyphenols, alkaloids, terpenes, and polysaccharides. The review transcends the limitations of a single compound category, systematically comparing the application effects of DES-combined techniques across these four major classes of natural products, identifying commonalities and specific differences across categories. For example, polyphenol extraction favors acidic DESs with strong hydrogen bond donors combined with ultrasound or microwave; alkaloid extraction relies on halogen-containing HBAs to utilize electrostatic interactions; terpene extraction requires hydrophobic DESs combined with ultrasound; polysaccharide extraction requires moderately viscous hydrophilic DESs, with enzyme assistance being particularly crucial.

## 2. Theoretical Basis and Rational Design of DESs for Natural Product Extraction

### 2.1. Interaction Mechanisms Between DESs and Target Components

The interactions between DESs and the bioactive constituents of natural products constitute the theoretical basis of effective extraction and mainly involve hydrogen bonding, electrostatic interactions, van der Waals forces, and π–π stacking [18].

Three parameters define the geometry of hydrogen bonding: distance, angle, and polar bond length. Short distances and favorable angles correspond to higher bond energies and stronger hydrogen bonds. In studies on anthocyanin extraction with Bet–LA and Bet–CA DES systems, molecular docking confirmed that the Bet–LA system forms shorter hydrogen bonds with lower binding energies, implying stronger interactions [19]. Molecular dynamics simulations have shown that caffeic acid (a model phenolic compound) forms, on average, more hydrogen bonds in Pr–LA than in water or 50% ethanol, accounting for its enhanced solubility in DESs [20,21].

Van der Waals interactions are short-range, non-directional attractions that typically occur between neutral molecules or atoms. They constitute one of the binding forces in hydrophobic deep eutectic solvents (HDESs) [5]. HDESs contain at least one poorly water-soluble component [22]. HDESs composed of neutral components exhibit only modest melting-point reductions and relatively low viscosity [23]. Depending on the nature of the HBA, HDESs are classified as ionic or non-ionic [22]. Early formulations combined decanoic acid as the HBD with various quaternary ammonium salts as HBAs [23]. Studies report that lengthening the HBA carbon chain increases hydrophobicity and promotes phase separation [22], thereby improving the extraction efficiency of volatile fatty acids [24]. The thymol–decanoic acid HDESs possess a weak hydrogen-bond network, enabling interactions with non-polar terpenoids through van der Waals forces and achieving a menthol solubilization capacity of 85 mg/mL [25].

Electrostatic interactions—the Coulombic attractive or repulsive potential energies between charged particles—constitute a distinct class of non-covalent forces. They are particularly important in alkaloid extraction: for example, Cl^−^ ions in ChCl–glycerol DESs form electrostatic attractions with alkaloid cations, and a boldine extraction yield of 2.362 mg/g—several times higher than that of methanol extraction—has been reported [17]. Similarly, electrostatic attraction proved indispensable in the extraction of evodiamine from *Evodia rutaecarpa* using an La–Gly–K_2_HPO_4_/ethyl acetate biphasic system [26].

π–π stacking occurs between aromatic-ring-containing species: for example, the aromatic rings of phenylalanine–urea DESs form π–π stacking interactions with the benzene rings of polyphenols, increasing gallic acid extraction by 25% [18].

Moreover, viscosity has been found to be positively correlated with the strength of hydrogen bonds. Highly viscous DESs (e.g., ChCl–glucose, 1200 mPa·s) offer strong solubilization but poor mass transfer, whereas low-viscosity DESs (e.g., ChCl–ethylene glycol, 200 mPa·s) provide efficient mass transfer but weaker solubilization [5]. Molecular dynamics simulations indicate that the interaction energy between DESs and target components is governed by the nature of the hydrogen bond acceptor (HBA) and hydrogen bond donor (HBD). For example, urea-based DESs exhibit stronger hydrogen bonding and higher solubilization capacity for polar compounds than glycerol-based DESs [18]. These interaction mechanisms not only explain the structural basis of DES extraction efficiency but also provide theoretical guidance for designing DESs tailored to different natural products.

### 2.2. Mechanism-Guided DES Design for Target Compound Classes

The tunability of DESs enables the effective extraction of specific target compounds through optimized HBA/HBD combinations. The design strategies outlined below link target compound properties to DES component selection, polarity, and viscosity, based on the interaction mechanisms described in Section 2.1.

Polyphenolic compounds. For the extraction of polyphenolic compounds (e.g., flavonoids, phenolic acids), DESs with strong hydrogen-bond-donating capacity should be selected, such as ChCl–citric acid DESs (1:3 molar ratio). The multiple carboxyl groups of citric acid can form extensive hydrogen bonds with polyphenols, affording a total phenolic yield of 103.37 mg GAE/g DW—markedly higher than that of ethanol extraction (62.1 mg GAE/g DW) [27]. Grape pomace is rich in polyphenolic bioactive compounds, and Jurić et al. [28] screened a series of natural deep eutectic solvents (NADES) for the extraction of different polyphenols from this matrix. The highest anthocyanin extraction was achieved with ChCl:1,2-propanediol (1:1) or ChCl:LacA:1,2-propanediol (1:1:1). This performance arises because anthocyanins are positively charged, highly polar, glycosylated, and sensitive to both pH and oxidation: Cl^−^ provides counter-anion interactions, while the hydroxyl groups of 1,2-propanediol form multiple hydrogen bonds with the glycosidic moieties, thereby enhancing extraction efficiency. Because anthocyanins are acid-sensitive, strongly acidic conditions (pH < 1) degrade their structures; a weak acid such as lactic acid provides a mildly acidic environment while suppressing hydrolysis of the anthocyanin glycosidic bonds. Ternary NADESs containing lactic acid have proved effective in extracting quercetin, attributed to structural similarities between the functional groups of quercetin and lactic acid. This indicates that distinct DES formulations are required for different polyphenol subclasses, depending on their specific physicochemical properties, and that the simultaneous extraction of multiple compounds necessitates joint optimization.

Alkaloids. For alkaloids (e.g., boldine, caffeine), DESs capable of electrostatic interactions should be selected. For example, the H^+^ in ChCl–hydrochloric acid DESs can protonate alkaloids, enhancing their solubility in DESs and affording a caffeine extraction yield of 1.76 mg/g [25].

Terpenoids. For terpenoids (e.g., menthol, limonene), hydrophobic DESs are more effective: thymol–lauric acid DESs (1:2 molar ratio) exhibit comparable performance and afford a menthol extraction yield of 85 mg/g [25].

For the extraction of biomacromolecules such as polysaccharides, DESs with moderate viscosity (approximately 500 mPa·s) have proven to be optimal. Although DESs with excessively low viscosity (e.g., ChCl–ethylene glycol, 200 mPa·s) offer high mass transfer efficiency, their weak hydrogen-bonding networks result in insufficient solubility; conversely, DESs with excessively high viscosity (e.g., ChCl–glucose, 1200 mPa·s) exhibit reduced extraction efficiency owing to mass transfer limitations. ChCl–urea (1:2, 500 mPa·s) afforded a 25.6% recovery in fucoidan extraction, reflecting a balance between solubility and mass transfer efficiency in the medium-viscosity range [10]. Furthermore, the water content of DESs can modulate their polarity and viscosity: for instance, increasing the water content of ChCl–1,4-butanediol DESs from 0% to 25% reduced their viscosity from 1500 mPa·s to 300 mPa·s, while boosting flavonoid extraction from 80 mg/g to 116.78 mg/g [29]. Response surface methodology has been used to optimise DES composition: for example, targeting grape pomace polyphenols with an optimized ChCl–malic acid DES (1:4 molar ratio) afforded a total phenolic yield of 92.23 mg GAE/g DW and a DPPH scavenging rate of 94.32% [27]. These strategies provide practical guidance for the targeted design of DESs, enabling the efficient extraction of diverse natural products.

The flexibility of DES design is also reflected in process optimization. Several key parameters must be optimized to achieve high-efficiency DES extraction of bioactive compounds, including the molar ratio of DES components, liquid/solid ratio, water content, extraction time, and auxiliary methods. In a study optimizing polyphenol extraction from *Moringa oleifera* leaves, the DES composition and water content were optimized to afford a formulation of 30% sweetener, 30% citric acid, and 40% water, which exhibited minimal viscosity and suitable acidity for polyphenol extraction [30]. Multiple studies confirm that water content is a critical process parameter. Excess water interferes with interactions among the components and collapses the hydrogen-bond network, making both the DESs and the co-existing phases more fluid [31]. This minimizes the effective contact between DESs and the matrix, thereby reducing extraction efficiency. Water content is a key parameter governing DES viscosity. As noted above, viscosity is determined by hydrogen-bonding network density. During extraction, hydrogen bonds exist among DES components, between DESs and target compounds, and between water and DESs [32]. At elevated water contents, the number of water–DES hydrogen bonds increases; excessive moisture (>50%) disrupts the DES hydrogen-bond network, leading to loss of eutectic properties and markedly reducing solubility. Therefore, an optimal water content range exists (typically 20–30%); within this window, the mass transfer enhancement from reduced viscosity outweighs solubility loss. Although these strategies provide a preliminary framework for matching DES properties with compound classes, they remain semi-empirical. Water content, temperature, and raw material matrix significantly modulate extraction performance, necessitating experimental optimization under the proposed guidelines.

## 3. Analysis of Synergistic Mechanisms Between DESs and Assistive Technologies

### 3.1. DES–Ultrasound Synergy: Cavitation Effect and Solvent Activation

**Figure 1:**
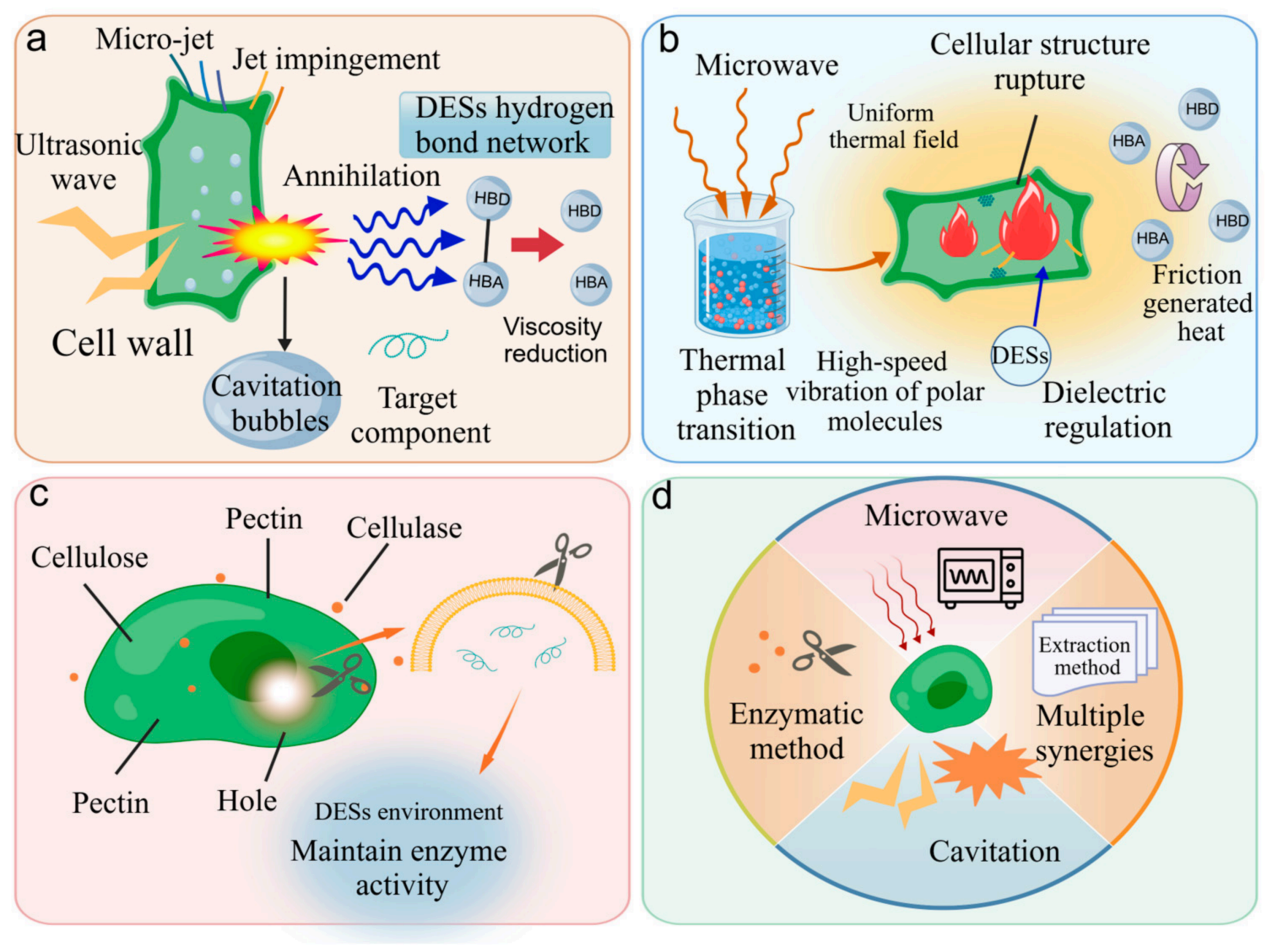
Schematic diagram of the synergistic mechanisms of DESs with auxiliary technologies: (**a**) DES–ultrasound (cavitation → viscosity reduction → enhanced mass transfer); (**b**) DES–microwave (volumetric heating → dipole rotation → selective absorption); (**c**) DES–enzyme (cell wall hydrolysis → pore formation → enhanced enzyme–substrate contact); (**d**) multi-technique synergy.

The synergism of the DES–ultrasound system arises from the combination of ultrasonic cavitation effects and the solvent properties of DESs (Figure 1a). Ultrasonic cavitation generates localized high temperatures and pressures, microjets, and shock waves that disrupt plant cell walls, thereby increasing the contact area between DESs and intracellular components. Meanwhile, cavitation can activate the hydrogen-bond network of DESs, locally reduce viscosity, and accelerate diffusion and solubilization [6]. As an example, in the DES-ultrasound synergistic extraction of rosmarinic acid from calendula, an extraction yield of 39.85% was obtained under 240 W ultrasonic power and a 35 min extraction time, representing a 50% improvement over DES-only extraction [7]. In the extraction of flavonoids from *Sophora japonica* flowers, ultrasonic cavitation increased the diffusion coefficient of DES from 1.2 × 10^−9^ to 3.5 × 10^−9^ m^2^/s, raising the flavonoid yield from 80 to 116.78 mg/g [29]. Furthermore, ultrasonic cavitation reduces DES viscosity: for example, the viscosity of ChCl–glycerol DES decreased from 1200 to 500 mPa·s under sonication, doubling the mass transfer efficiency [6]. In rapeseed protein extraction, two NADES systems—ChCl:Glu (1:1) and ChCl:water—were compared with and without ultrasonication; ultrasonic treatment increased the protein extraction rate by 10.4% and 12.6%, respectively. In the ChCl:Glu (1:1) group, cavitation microjets disrupted cell walls and disintegrated protein aggregates, and ultrasound-induced localized heating further enhanced protein solubility. In the aqueous group, the high water content and low viscosity lowered the cavitation threshold, intensifying bubble collapse and consequently increasing solubility [33]. The design versatility of DESs further enhances ultrasonic synergy: for example, in ultrasound-assisted liquid–liquid microextraction with thymol–decanoic acid DESs, the hydrophobicity of the DES was coupled with ultrasonic cavitation, lowering the detection limit of polycyclic aromatic hydrocarbons to 0.0039–0.0098 μg/L [25]. In sturgeon skin collagen extraction, extraction times of 5, 10, 15, and 20 min were compared; 10 min proved optimal, affording a 21% relative increase in extraction rate, whereas at 20 min the yield decreased owing to sample degradation caused by prolonged mechanical shearing and localized heating [34]. Molecular dynamics simulations predict that ultrasonic cavitation can disrupt the hydrogen-bonding network of DESs, thereby potentially reducing viscosity [5]. However, direct experimental visualization of this phenomenon at the molecular scale remains limited; the observed viscosity reduction (e.g., ChCl–glycerol, from 1200 to 500 mPa·s) has been measured macroscopically under sonication, and the extent to which this arises from hydrogen-bond disruption rather than thermal effects or bubble-induced mixing remains to be elucidated through in situ characterization. Overall, the DES–ultrasound system enhances mass transfer and activates solvent properties through cavitation, enabling efficient extraction of natural products; however, excessive sonication can degrade target components through mechanical shearing and thermal effects, so ultrasonic parameters must be optimized.

### 3.2. DES–Microwave Synergy: Volumetric Heating and Selective Absorption

The core advantage of the DES–microwave (MAE) synergistic extraction system lies in the so-called volumetric heating induced by microwaves (Figure 1b). Microwaves are absorbed directly by the polar molecules of the DESs and the matrix, producing rapid and uniform heating with reduced temperature gradients, which is favorable for heat-sensitive compounds. In addition, the dielectric properties of DESs can be tuned through the choice of HBA/HBD to achieve selective microwave absorption [6]. Microwave heating also reduces the viscosity of some DESs, thereby improving mass transfer. For example, in DES–microwave synergistic extraction of polyphenols from grape pomace, 800 W microwave power for 5 min achieved extraction equivalent to 120 min of conventional heating, with polyphenol recovery enhanced by 20% [16]. When microwaves were applied to the extraction of flavonoids from *Sophora japonica* flowers, volumetric heating tripled the temperature uniformity of the DESs and increased the flavonoid yield threefold (from 80 to 116.78 mg/g) [29]. Vidić et al. [35] combined four self-developed NADESs with 24 s pulsed microwave irradiation to simultaneously isolate 11 phenolic acids and flavonoids from willow bark and leaves, with parallel controls using microwave-only purified water and 48 h ethanolic maceration. The DES-MAE synergistic extraction rate exceeded those of MAE alone and conventional organic solvent extraction. This approach not only maximized extraction efficiency but also shortened microwave exposure and reduced damage to the extracts. Tuning the dielectric constant of DESs enhances synergistic interaction with microwaves: ChCl–lactic acid DESs reportedly exhibits a higher dielectric constant (~45) than ChCl–glycerol DES (~30). If verified, this property would favor microwave absorption; however, the boldine extraction yield of 2.362 mg/g reflects the combined effects of dielectric properties, acidity-driven protonation, and other factors, and thus cannot be attributed solely to microwave absorption efficiency [17]. Moreover, microwave synergy can reduce DES viscosity: for example, the viscosity of ChCl–1,4-butanediol DESs was reduced to 800 mPa·s under microwave irradiation, improving mass transfer efficiency 1.5-fold [29]. Infrared thermography showed that the temperature uniformity of the DES–microwave system reached 95%, far exceeding the 70% achieved by conventional heating [6]. These mechanisms enable the DES–microwave system to extract natural products rapidly and efficiently through volumetric heating and selective absorption.

### 3.3. DES–Enzyme Synergy: Biocatalysis Coupled with Green Solvents

DES–enzyme synergistic extraction combines the catalytic specificity of enzymes with the green solubilizing capability of DESs. The key task is to identify enzymes that are compatible with DESs and retain high activity (Figure 1c). The mild DES environment preserves enzymatic activity, while the selective enzymatic degradation of cell walls facilitates DES penetration, thereby coupling biocatalysis with green solvents [36]. Enzymes selectively degrade cell wall constituents (cellulose, pectin, hemicellulose, etc.), markedly reducing mass transfer resistance and allowing DESs to penetrate and solubilize target compounds more readily. For example, in DES–enzyme synergistic extraction of pectin from apple pomace, the pH of ChCl–citric acid DESs (5.5) matched the optimum of pectinase, whose activity was retained at 90%, increasing the pectin yield by 15–32% [9]. In the extraction of brown-algal polysaccharides from kelp, the mild conditions of ChCl–urea DESs preserved 85% of cellulase activity, enhanced enzyme efficiency by 40%, and afforded a polysaccharide yield of 25.6% [10]. Using a DES pretreatment–enzymatic hydrolysis strategy, ChCl:Gly pretreatment of rice straw cellulose raised the glucose yield to 86%, leaving only ~14% of the cellulose unhydrolyzed [37]. Enzymatic treatment of bamboo alone is ineffective, whereas pretreatment with a ChCl:FA system markedly enhances enzymatic performance, increasing the glucose yield to 76 and the ethanol concentration to 13.4 g/L [38]. The design flexibility of DESs can further improve enzyme synergy: for example, a ChCl–glycerol DES with a viscosity of 500 mPa·s permits enzyme mobility while providing a sufficient hydrogen-bonding milieu, affording 95% retention of lipase activity [36]. For lipase in ChCl–1,4-butanediol DESs, a water content of 0–20% supported higher enzyme activity than 50–90%. This observation likely reflects the competing effects of water as a lubricant enabling protein dynamics at low concentrations versus a hydrogen-bond disruptor that destabilises the DES network at high concentrations. Whether this optimal water content range extends to other enzymes or DES formulations has not been established [36]. Tan and Dou reported that molecular dynamics simulations of lipase in ChCl–glycerol DESs suggested hydrogen bond interactions between the solvent and enzyme surface residues. Whether this effect stabilizes enzyme conformation in other DES–enzyme systems, or whether the observed activity retention (95% for lipase) primarily reflects the solvent’s water content and pH rather than specific hydrogen bond stabilization remains to be determined across a broader range of biocatalysts [36]. Because enzymes are readily deactivated under acidic conditions, DESs containing acidic components cannot be used in enzymatic processes. Non-acidic DES constituents such as glycerol or urea can preserve full enzyme activity and even enhance it [39]. These mechanisms enable the DES–enzyme synergistic system to extract natural products efficiently and selectively by coupling biocatalysis with green solvents. DES–enzyme cooperative processing is also widely used to break down water-insoluble cellulosic materials, converting them into fermentable sugars and bioethanol. This approach reduces resource waste and landfill volumes and lowers greenhouse gas emissions, making it environmentally favorable [40,41].

### 3.4. Triple and Multiple Synergistic Technologies

Combining DESs with two or more auxiliary technologies integrates their respective advantages to achieve ultra-high extraction efficiency (Figure 1d). Triple and multiple synergistic technologies improve extraction performance by coupling DESs with physical fields and enzymes. For example, in the synergistic extraction of rosmarinic acid combining DES–ultrasound and enzymatic techniques, ultrasonic cavitation enhanced enzyme–substrate interactions by 40%, while the high solubilizing capacity of DESs afforded a rosmarinic acid yield of 39.85%, far exceeding single-technology processes [7]. In the DES–microwave–enzyme synergistic extraction of grape pomace polyphenols, microwave volumetric heating accelerated enzymatic reactions while DESs promoted selective absorption, affording a total phenolic content of 103.37 mg GAE/g DW and an antioxidant activity of 101.85% [27]. Moreover, DES–ultrasound–microwave synergistic extraction of *Sophora japonica* flowers combined ultrasonic cavitation with microwave volumetric heating, increasing the flavonoid yield from 116.78 to 132.5 mg/g [29]. Integrating NADESs with microwave–ultrasonication raised the pectin yield of dragon fruit peel to ~19.4%, compared with ~7% obtained by conventional acid extraction, while maintaining high pectin quality; microwave pretreatment first disrupted the cell walls, and subsequent ultrasonication released the loosened material [42]. These multi-technique synergies combine the strengths of the individual methods, drastically shortening extraction times while increasing the yield and purity of target compounds severalfold, and thus represent a promising route for the ultra-efficient industrial extraction of natural products.

It should be noted, however, that the term “synergy” as used in the aforementioned studies predominantly reflects phenomenological yield enhancement rather than mechanistically validated synergy. True synergy is defined as a combined effect exceeding the arithmetic sum of independent contributions (SC > 1, where SC = Y_combined/(Y_A + Y_B)), whereas many reported cases may represent merely additive effects (SC ≈ 1) of independent mechanisms operating in parallel. The absence of essential independent control groups (DESs alone, auxiliary technology alone, and combined treatment) and quantitative synergy metrics in most studies precludes definitive mechanistic interpretation. Future investigations should incorporate comprehensive factorial controls and kinetic modeling to distinguish synergistic interactions from apparent yield enhancement.

### 3.5. Comparison of DES–Ultrasound, DES–Microwave, DES–Enzyme, and Multi-Component Composite Systems

Deep eutectic solvents (DESs) have shown great potential as green solvents for natural product extraction. However, inherent limitations of DES-only systems—such as high viscosity that restricts mass transfer—represent major bottlenecks. Therefore, the synergistic combination of DESs with physical fields (ultrasound, microwaves) or biotechnology (enzymes) has become the mainstream direction of current research. The following provides a critical comparison of DES–ultrasound, DES–microwave, DES–enzyme, and multicomponent composite systems across several key dimensions (the DES–ultrasound, DES–microwave, and DES–enzyme pairings are hereafter abbreviated as UA-DES, MA-DES, and EA-DES, respectively):

#### 3.5.1. Extraction Efficiency and Selectivity

DES–ultrasound (UA-DES): High efficiency, but poses some risk to heat-sensitive substances. The ultrasonic cavitation effect effectively disrupts cell walls, significantly enhancing mass transfer rates. In the extraction of total flavonoids from red plum peel, ultrasound-assisted urea-based NADESs yielded the highest recovery (3884.98 mg CE/kg dm) [43], with its selectivity closely correlated with ultrasound power and duration. However, excessively long ultrasound durations or excessively high power may lead to localized high temperatures, causing the degradation of heat-sensitive compounds (such as anthocyanins) [44].

DES–Microwave (MA-DES): Extremely efficient with adjustable selectivity. The volumetric heating properties of microwaves rapidly reduce the viscosity of the DESs, greatly enhancing mass transfer. When extracting polyphenols from *Eleutherococcus senticosus* fruits, the combination of DESs and microwave technology yields a higher polyphenol recovery rate than either ultrasonication or microwave treatment alone [45]. By adjusting microwave power and duration, selective extraction of specific compounds can be achieved; however, excessive microwave irradiation may also lead to the degradation of active components such as polyphenols [44].

DES–Enzyme (EA-DES): Excellent selectivity, moderate efficiency. Enzymes (such as cellulase and xylanase) can selectively hydrolyze specific components in cell walls (such as cellulose and pectin), thereby promoting DES penetration. For example, when extracting flavonoids from hollyhock leaves, the enzyme-assisted DES system achieved a 77.1% higher yield compared to conventional solvent extraction [44]. However, its efficiency is highly dependent on enzyme activity, pH, and temperature, and the processing time is relatively long [46].

Multi-component composite systems: Offer the highest overall efficiency but are complex to optimize. By combining two or more auxiliary techniques (e.g., ultrasound–enzyme–DES, ultrasound–microwave–DES), a synergistic effect of “1 + 1 > 2” can be achieved. For example, a recent study increased the extraction yield of flavonoids from hollyhock leaves to 37.54 mg/g using ultrasound–enzyme-assisted DESs [44].

#### 3.5.2. Scalability and Industrial Feasibility

UA-DES: Relatively high; pilot-scale cases have been reported. The technology for scaling up ultrasonic equipment to industrial levels is relatively mature, but the uneven distribution of ultrasonic energy in large-scale reactors remains a major challenge [47].

MA-DES: Moderate; energy efficiency is key to widespread adoption. The rapid heating capability of microwaves offers significant advantages in shortening extraction time, and the high dielectric loss of DES enables DES to absorb microwave energy efficiently, theoretically resulting in lower energy consumption. However, the limited penetration depth of microwaves makes it difficult to process large batches of material, and equipment investment and energy costs require a thorough techno-economic analysis (TEA) before large-scale production can be implemented [47].

EA-DES: Low. High enzyme costs, sensitivity of enzyme activity to environmental conditions, and long processing times collectively limit the application of this strategy in continuous, large-scale industrial settings [46,47].

Multicomponent composite systems: Low. The introduction of multiple physical and biological variables (such as ultrasonic power, microwave power, enzyme type and dosage, temperature, and time) renders process optimization extremely complex and difficult to control; consequently, current research remains largely confined to the laboratory scale.

#### 3.5.3. Solvent Recovery and Energy Consumption

UA-DES: Recovery is moderately difficult, whereas energy consumption is low. Because no high temperatures are involved, thermal damage to the DES structure is minimal, thereby facilitating solvent recovery. However, the additional recovery steps (such as anti-solvent precipitation and membrane separation) still add to the overall process costs [46]. Notably, in the extraction of polysaccharides from *Polygonatum sibiricum*, the UA-DES method required far less extraction time (20.5 min vs. 60 min) and substantially lower energy consumption than the conventional ethanol reflux method, yet solvent recovery remained a major challenge [44].

MA-DES: Energy efficiency is a distinct advantage, but thermal stability must be considered during recovery. Microwave heating itself is highly efficient, significantly reducing energy consumption and processing time. However, the high-temperature conditions may alter the physicochemical properties of DESs, thereby compromising the reusability of the recovered solvent. A study on the extraction of limonin analogues from round pomelo showed that the DES recovery rate decreased from 86% to 71% after three cycles [44].

EA-DES: Energy consumption is low, but recovery is difficult. Enzyme-assisted processes are typically conducted under mild conditions, which keeps energy demand low. However, efficiently separating DESs from enzymes and recovering the enzymes without compromising their activity remains a pressing challenge [44].

Multicomponent composite systems: Both energy demand and solvent recovery remain problematic at the system level. Although the individual techniques are inherently energy-efficient, the combined process is complex and involves numerous control points, substantially increasing total operating costs; moreover, the recycling of solvents and enzymes becomes even more cumbersome.

#### 3.5.4. Processing Time and Product Stability

UA-DES: Short processing time and good stability. Processing typically ranges from a few minutes to tens of minutes. Ultrasonic disruption of cell walls also exerts a mild sterilization effect, helping to preserve extract stability.

MA-DES: Extremely short processing time, but with risks to heat-sensitive compounds. Extraction can be completed within a few minutes or even tens of seconds. For thermally stable compounds, bioactivity is well preserved owing to the brief exposure; however, heat-sensitive compounds remain prone to degradation.

EA-DES: Long processing time, with a risk of activity loss. Enzymatic hydrolysis typically takes several hours (e.g., 10 h) [46]; such prolonged processing may compromise the stability of bioactive compounds in the extract.

Multi-technique hybrid systems: Processing times are intermediate between those of UA-DES and EA-DES. The integration of ultrasound or microwave irradiation can significantly shorten the enzymatic hydrolysis time and improve overall efficiency [44].

In summary, a comprehensive evaluation across the above dimensions indicates that DES–ultrasound achieves the optimal balance among extraction efficiency, equipment maturity, and production cost, making it the preferred route for large-scale resource recovery at this stage. DES-microwave, with its extremely short processing time, is more suitable for small-batch crude extraction of hard-to-process feedstocks, whereas DES-enzyme, despite its long processing time, operates under mild conditions and is limited to the purification of high-value-added bioactive compounds. Multi-component hybrid systems offer the best extraction performance but face significant constraints in process control and equipment compatibility and currently remain at the pilot-scale development stage. This comparative framework provides researchers with a rational basis for technology selection and lays the analytical foundation for the discussion of process scale-up in Section 5.

## 4. Advances in the Application of DES Synergistic Technologies

### 4.1. Extraction of Polyphenols and Flavonoids

Polyphenols and flavonoids are vital plant bioactive compounds for which DES synergistic technologies have proven highly effective (see Table 1). As secondary metabolites, polyphenols and flavonoids share common structural features such as polyhydroxylated benzene-ring backbones. They are widely distributed in plants, where they function both in chemical defence and in signal regulation. Polyphenols are broadly defined as compounds bearing two or more phenolic hydroxyl groups and can be further classified into flavonoids, phenolic acids, lignols, and lactones. The phenolic OH groups on the aromatic rings endow them with hydrogen/electron-donating capacity. More than half of all polyphenols are flavonoids, most of which possess a characteristic 2-phenylchromenone skeleton (C6–C3–C6) in which rings A and B are connected by a three-carbon bridge (C-ring), forming an extended conjugated π system. Further hydroxylation, methoxylation, and glycosylation subdivide them into flavonols (quercetin, kaempferol), flavan-3-ols (catechin, EGCG), anthocyanins (cyanidin-3-O-glucoside), and isoflavones (genistein, genistin), comprising over 6000 derivatives.

#### 4.1.1. Polyphenols

Polyphenolic compounds exhibit diverse physiological activities. In a study on black rosehip polyphenols, Zannou et al. [68] employed a sodium lactate–acetate (LASA) system with DES-MAE; by adjusting the moisture content to 54%, the solid-to-solvent ratio to 1:45, and the microwave temperature to 54 °C, the total phenolic content reached 3020 mg GAE/g, a 50% improvement over conventional methanol extraction. UAE-DES extraction of polyphenols from ginseng leaves offers significant advantages over traditional organic solvent extraction in terms of efficiency and environmental friendliness. Hydrogen-bond networks form between DESs and the target compounds, and the low viscosity of the choline chloride–glycerol system matches the hydroxyl-rich structure of polyphenols, following the like-dissolves-like principle; coupling with ultrasonication further shortens the extraction time relative to conventional methods [69]. Choline chloride–propylene glycol (1:2.5) extraction of camellia seeds affords higher polyphenol recovery than traditional 80% aqueous methanol, while also enhancing the antioxidant activity of the extracts and preserving a more complete component profile [70]. The literature indicates that phenolic extraction efficiency depends on the nature of both HBA and HBD: choline-type HBAs are highly effective, and polyol- and organic acid-based HBDs outperform other HBD types [14]. For the extraction of polyphenols from date seeds, carboxylic acid-based HBDs likewise afforded higher extraction rates, consistent with previous studies [71,72,73].

#### 4.1.2. Flavonoids

Owing to their large share among polyphenols, flavonoids are discussed separately here. The DES-based strategies used for flavonoid extraction are similar to those for polyphenols. Wang et al. [74] isolated flavonoids from bilberry leaves using a choline chloride–lactic acid system coupled with microwave extraction, obtaining a total flavonoid yield of 305.82 mg/g—significantly higher than that of traditional ethanol extraction. DES–ultrasonic extraction of polyphenols from *Artemisia capillaris* afforded higher extraction efficiency, richer chemical composition, and greater antioxidant activity than conventional 48% ethanol extraction [75]. For the extraction of flavonoids from male *Eucommia ulmoides* flowers, choline chloride–oxalic acid DESs coupled with microwave-assisted extraction (MA-DES) afforded a more than 30-fold higher yield with shorter processing time than ethanol, microwave, or DES extraction alone [76].

Anthocyanins, a subgroup of flavonoids, serve as natural pigments in foods and cosmetics and also exhibit antioxidant activity [77]. However, anthocyanins are highly unstable, and extraction conditions strongly affect their stability [15]. DES-based extraction is an emerging technique for anthocyanins: owing to their tunability, DESs provide an extraction medium that better preserves the structural integrity and functionality of anthocyanins. In the NADES extraction of butterfly pea anthocyanins, the ChCl:Gly system afforded better storage stability than pure-water extraction: losses after 21 days of refrigeration were less than half of those in the aqueous system, and antioxidant activity was better retained [78].

Moreover, DES synergistic technologies improve the stability of polyphenols: for example, polyphenols obtained from grape pomace by DES–microwave synergistic extraction retained 85% of their activity after 6 months of storage at 4 °C, compared with only 60% for ethanol extraction [16]. These examples demonstrate that DES synergistic technologies not only enhance the rate and efficiency of polyphenol and flavonoid extraction but also improve product stability, thereby offering an economically viable route toward their medicinal application.

### 4.2. Extraction of Alkaloids

**Figure 2:**
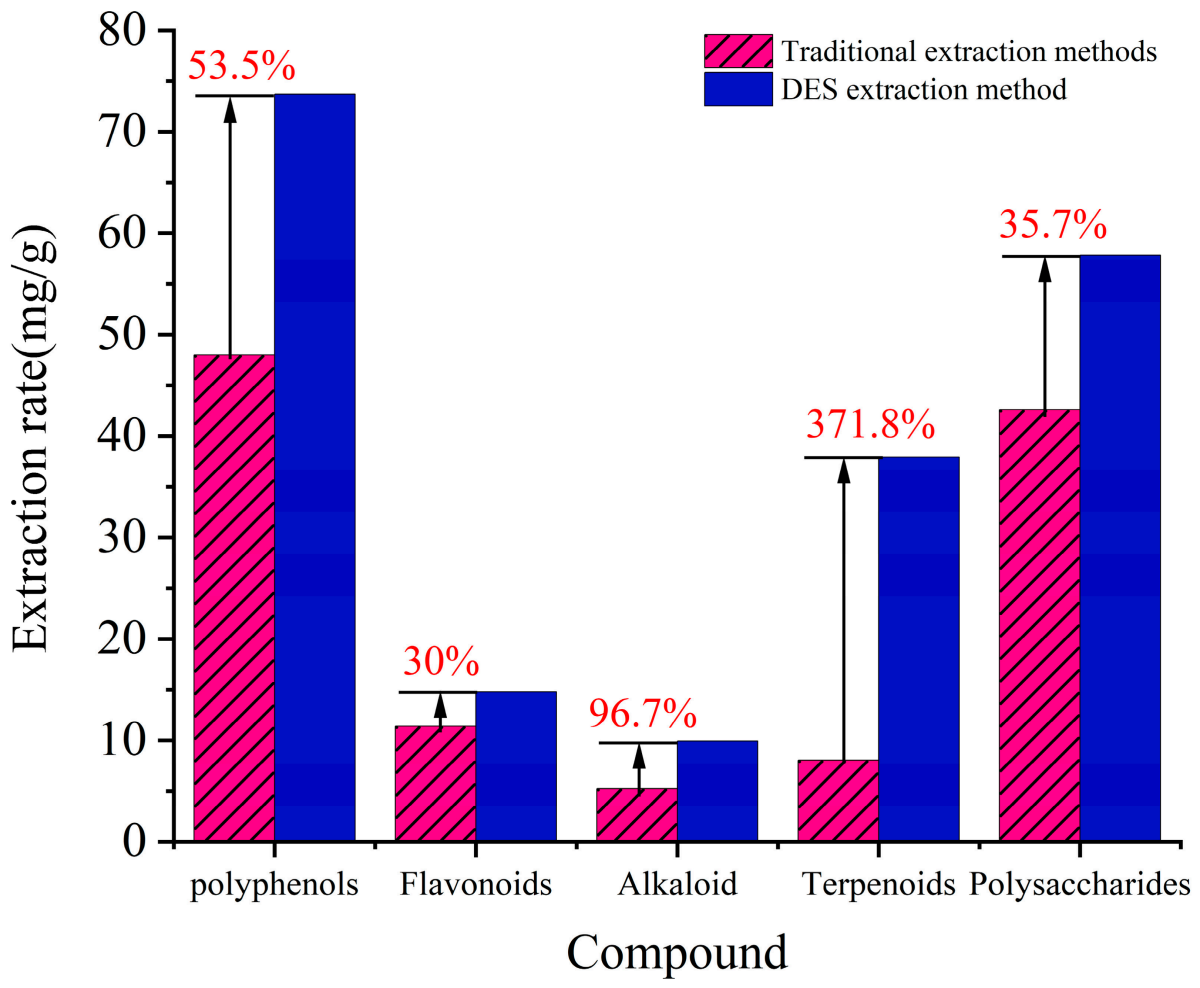
Comparison of bioactive compound yields obtained by DES-based methods versus traditional methods within individual studies [76,79,82,83]. Note: Each pair of bars represents data from a single study under consistent experimental conditions (same raw material, extraction temperature, and analytical method), enabling internal comparison but not cross-study benchmarking.

DESs exhibit high solubilizing capacity and selectivity toward alkaloids. Acidic DESs or DESs bearing specific anions can efficiently extract alkaloids through ion-exchange or ion-pairing processes. Alkaloids are a family of nitrogen-containing natural bases, most of which are derived from plant amino acid metabolism. DES synergistic technologies exhibit high selectivity and efficiency in their extraction. Using a ChCl–urea system to modify Fe_3_O_4_/molecularly imprinted polymers (MIPs) for the extraction of theobromine and theophylline from green tea, purification rates were reported to be more than 30 percentage points higher than those of other systems [79] (Figure 2). Under optimized conditions, a biphasic system of a low-melting-point solvent and ethyl acetate (DES-EA) afforded an alkaloid yield of 757.71 µg/g from *Evodia* fruit residue, significantly higher than that of the traditional methanol method (110.22 µg/g). Thermodynamic and kinetic studies suggest that EA and the alkaloids form only weak complexes, allowing facile separation of products and solvent by vacuum distillation [26]. Moreover, DES synergistic technologies enable selective extraction with minimized impurity levels: for example, combining choline chloride–ethylene glycol (1:2) with ultrasonication for the extraction of Stephania root alkaloids provided a dual solvent–mechanical driving force that doubled the extraction efficiency and shortened the processing time relative to conventional approaches [80]. Various studies indicate that acid–base NADESs are frequently employed for alkaloid extraction because they can form salts with the alkaloids, thereby facilitating their recovery [14]. Screening of 75 DESs against five alkaloids showed that acidic DES-bearing–COOH groups afforded higher extraction rates for most alkaloids than neutral or sugar-based DESs. Strongly basic alkaloids form salts with the acidic counterparts of DESs, which increases their water solubility [81].

### 4.3. Extraction of Terpenes and Essential Oil Components

Hydrophobic DESs offer unique advantages for the extraction of lipophilic terpenes and essential oil components. The polarity of DESs can be tailored to enable the targeted extraction of terpenes with different polarities. Moreover, in situ DES formation with essential oil components followed by phase separation provides a new route for the effective separation of volatile oils. Terpenes and volatile oil components (e.g., menthol, limonene) possess characteristic aromas and pharmacological properties. DES-assisted technologies achieve high retention of these volatiles with low energy consumption during extraction. Supercritical carbon dioxide (SC-CO_2_) extraction combined with NADES–ultrasonic extraction is an efficient technique for isolating bioactive compounds, including terpenes, from citrus peels. Its low operating temperature—lower than that of other extraction methods—makes it especially suitable for heat-sensitive, volatile monoterpenes, sesquiterpenes, and their derivatives [84]. Tan Phat Vo et al. improved terpenoid extraction efficiency fourfold over conventional methods using a two-step process—high-shear enzyme-synergistic cell wall disruption followed by DES-mediated hydrogen-bond network formation with the terpenoids [82] (Figure 2).

Plant extracts frequently contain essential oils (volatile oils), which are rich in terpenes and are usually removed by pretreatment before extraction. He et al. [85] developed in situ DES formation with essential oils as a new technique. Because terpenes form no hydrogen bonds with the DES, they are retained in the upper layer after cooling and phase separation. Water can then be used to gently disrupt the DES, enabling back-extraction. Chen et al. combined DES pre-soaking with ultrasonic water distillation, increasing the extraction yield 3.3-fold. Studies suggest that highly polar DESs may oxidize terpenes, whereas less polar DESs are more suitable for extracting lipophilic hydrocarbon terpenes [86].

### 4.4. Extraction of Polysaccharides

In polysaccharide extraction, DESs provide an ideal dissolution environment that, unlike strong acids or bases, does not damage glycosidic bonds. DES-assisted extraction achieves high purity and high yields of these compounds. In the extraction of star anise polysaccharides, DESs demonstrated their tunability across different polarities. DESs combining an HBA (e.g., citric acid) with polyol HBDs exhibit mild acidity, avoiding the harsh acid-induced degradation of glycosidic bonds. DES–microwave synergy shortened the extraction time and afforded a 5.14% yield [87]. In DES–ultrasound synergistic extraction of starch from corn, ChCl–glucose with 300 W ultrasonic power afforded a starch extraction rate of 82%, significantly higher than that of conventional water extraction (55%) [37]. Mahmoud et al. found that sulfated polysaccharides, owing to their sulfate groups, exhibit stronger acid dependency. Adding carboxylic acids to binary DESs reduced the structural stability of sulfated polysaccharides, thereby improving their solubility [88]. Microwave–DES extraction of *Dendrobium officinale* polysaccharides showed that microwave irradiation disrupts cell walls by acting on polar molecules, accelerating polysaccharide dissolution while better preserving antioxidant properties [89]. Loquat leaf polysaccharides obtained by DES–ultrasonic co-extraction yielded 57.8 mg/g, versus 42.6 mg/g by traditional methods (Figure 2), with lower average molecular weight and higher antioxidant activity [83]. These cases (Table 1) demonstrate that DES synergistic extraction technologies achieve both high purity and high yield of polysaccharides, providing an effective route for their development into functional products.

## 5. Current Challenges and Future Outlook

### 5.1. Core Challenges for Industrial Scale-Up

Although DES synergistic technologies have performed exceptionally well at the laboratory scale, numerous challenges remain for industrial scale-up [90].

1. Cost and recovery of solvents: Some raw materials for DES preparation are expensive [90], and effective, energy-efficient solvent recovery and recycling technologies are not yet fully mature. The efficiency of DES recycling progressively declines with repeated use: acidic DESs corrode equipment, whereas alkaline DESs exhibit increased viscosity and reduced heat transfer coefficients during cycling [91].

2. Compatibility of equipment and process scales: DESs have intrinsic limitations, the most prominent being their high viscosity. High-viscosity DESs place special demands on transportation and mixing equipment. Achieving uniform energy distribution in scaled-up physical-field technologies (ultrasound, microwave) is challenging, and the multi-factor coupled process parameters must be re-optimized during scale-up. Together, these factors currently constrain industrial adoption.

3. Toxicological Data and Regulatory Gaps: DES solvents are generally considered to be green solvents, but their toxicological information is incomplete. The lack of harmonized, quantitative green certification represents one challenge to DES use in the food and pharmaceutical industries [92]. Food-grade DESs predominantly comprise choline chloride or NADESs formed from organic acid, polyol, or sugar constituents, which generally exhibit favorable biodegradability. However, not all DESs are readily biodegradable, and their biodegradability depends strongly on component selection. Thermal or photochemical degradation can generate byproducts whose bioactivities may differ substantially from those of the parent DESs. The environmental fate of DESs in aquatic systems remains poorly characterized, and standardized ecotoxicity assays across multiple trophic levels are lacking. Consequently, dedicated regulatory frameworks for DESs have not yet been established, leaving significant regulatory gaps.

4. Insufficient fundamental data: Reports indicate that only ~10% of published DES studies provide complete solid–liquid phase diagrams, viscosity–temperature relationships, and correlations between ΔfusH and pKa [93]. The parameters of DES synergistic technologies (e.g., ultrasonic power, microwave power, enzyme concentration) must be optimized through multi-factor designs. During industrial scale-up, parameter fluctuations are likely to reduce extraction rates [90]. Static electricity generated during extraction, as well as pipeline blockage, poses additional challenges. Independent regulatory frameworks for DESs have not yet been established, leaving significant regulatory gaps.

Regarding solvent recovery, commonly used methods include antisolvent precipitation, membrane separation, and low-temperature recrystallization. Antisolvent precipitation often requires further post-treatment. Membrane separation offers high recovery efficiency; however, the high viscosity of DESs increases membrane fouling and the required operating pressure. Exploiting the low melting points of DESs, low-temperature recrystallization enables regeneration; however, the high solubility of choline chloride in aqueous systems makes complete crystallization difficult. The regeneration efficiency requires further optimization under specific experimental conditions, and after recrystallization, DES components may need to be replenished to restore the original composition [94]. Some studies have proposed CO_2_-responsive DESs for solvent recovery; experimental results showed that after three recovery cycles, the extraction yield decreased from 30.33 ± 1.60 mg/g in the first cycle to 19.58 ± 0.96 mg/g by the third cycle (a reduction of approximately 35%) [95]. In addition, DES recovery can be achieved through pH-regulated phase separation, which has been experimentally demonstrated to permit more than five reuse cycles [96]. Regarding energy consumption, ultrasonic and microwave treatments generally consume less energy than conventional methods. Although DES raw materials and processing equipment are relatively expensive, conventional methods often require multiple purification steps and generate more environmentally harmful waste than DES-based processes. The viscosity of high-viscosity DESs can be readily lowered by adding a moderate amount of water, and moderate heating during extraction can further enhance mass transfer.

### 5.2. Cutting-Edge Directions and Future Outlook

**Figure 3:**
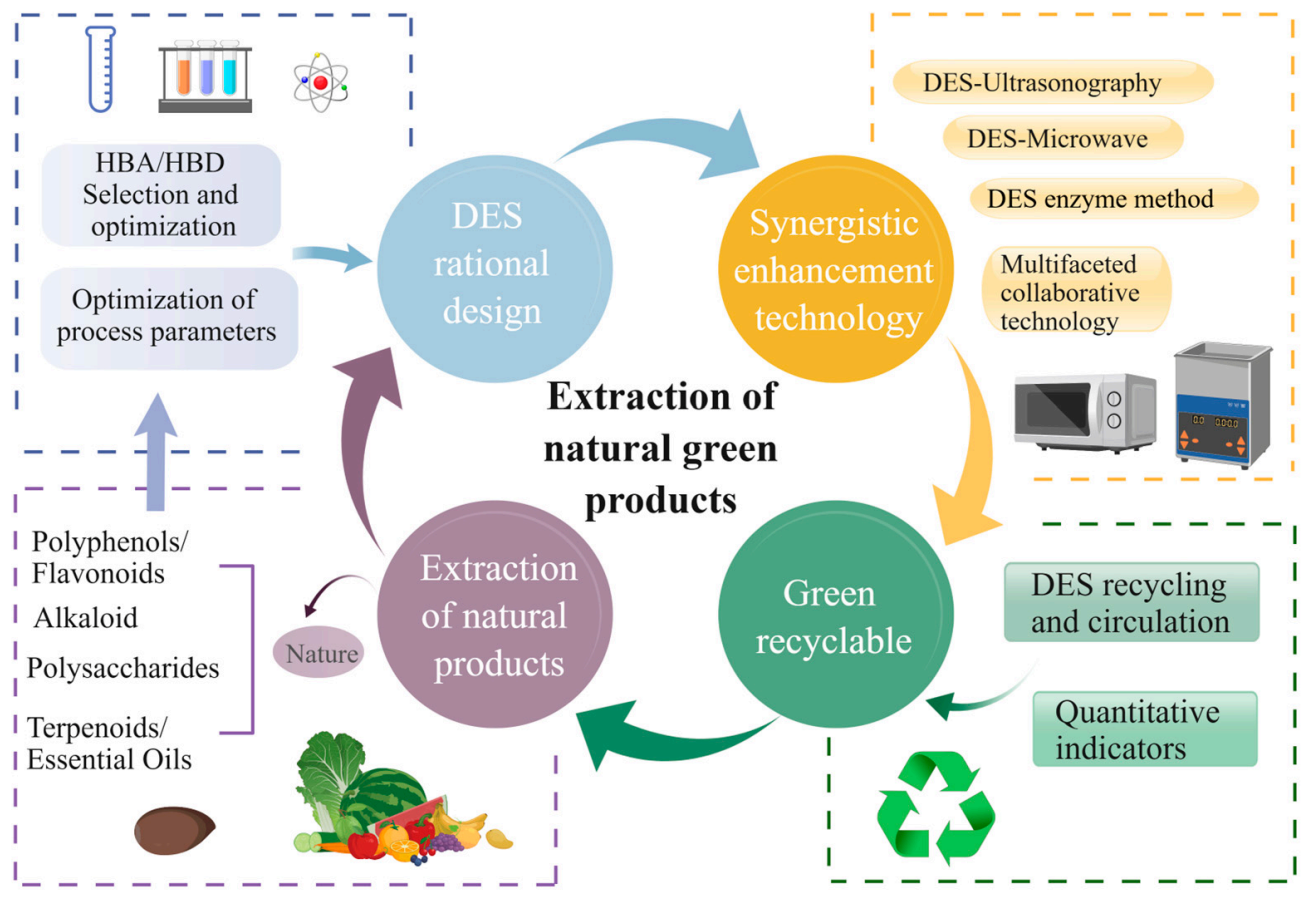
The general process of extracting bioactive substances using DESs.

To overcome the above challenges, it is essential to clarify future directions for DES development and integration with emerging technologies. Figure 3 illustrates the general process of extracting natural bioactive compounds with DES. Future-oriented directions for DES synergistic technologies include intelligent DES design, continuous extraction processes, multifunctionalization of synergistic methods, mechanistic studies, and the expansion of application scope [97].

1. Intelligent DES design: Machine learning and molecular modeling tools (e.g., COSMO-RS) are used to predict the physicochemical properties of DESs and their interactions with target components, enabling the targeted development of high-performance DESs. For example, a random forest model predicted the solubilizing capacity of DESs toward polyphenols with 90% accuracy [98]. COSMO-RS has been experimentally validated as a DES screening tool, but its independent predictive accuracy is limited. Therefore, COSMO-RS is currently better suited as a preliminary screening tool to narrow the experimental scope, rather than as a substitute for experimental validation.

2. Continuous extraction processes: Continuous and automated DES synergistic extraction equipment and processes should be developed to achieve efficient, stable, and energy-efficient large-scale production. An ultrasonic continuous extraction unit (DES–ultrasonic) with a throughput of 100 kg/h can maintain extraction rates consistently above 85% [6]. In multi-omics-guided optimization of extraction conditions, metabolomics is used to analyze the compositional distribution of natural products and thereby optimize DES structures. For example, a dual-functional DES (a blend of ChCl–citric acid and thymol–lauric acid) was designed to simultaneously extract flavonoids and terpenoids from ginkgo leaves [99]. However, the aforementioned studies mostly focus on traditional solvents or non-DES systems, and data on the design, long-term operational stability, and economic viability of DES-specific continuous flow reactors remain insufficient, requiring further pilot-scale validation.

3. Multifunctional implementation of DES synergistic technologies (e.g., integrated extraction–separation) is a future trend. For example, in a DES–membrane separation synergistic system, membrane separation purified the extract while achieving 90% DES recovery and 95% target-compound purity [6]. However, these designs currently remain at the laboratory validation stage, and the phase separation efficiency, membrane fouling control, and long-term cyclic stability during industrial scale-up still require systematic evaluation.

4. Mechanistic research and standardization: In situ analytical methods should be applied to elucidate synergistic mechanisms, and toxicity assessment systems and standardized DES databases should be developed to strengthen regulation.

5. Expanding the scope of applications: DES synergistic technologies should be explored for the extraction of a broader range of bioactive compounds, such as proteins, nucleic acids, and lipids, and for additional functional uses in biocatalysis and materials synthesis. Beyond extraction, the downstream application of DES extracts is becoming an emerging research focus, including the use of DES/NADES extracts for antioxidant and colour stabilization in functional foods and beverages, the incorporation of DES polyphenol extracts into biopolymer-based active packaging films, and the use of NADESs as delivery carriers to enhance the bioavailability of bioactive compounds. These application-oriented studies are expected to bridge DES synergistic extraction technology and industrial utilization; however, issues such as extract stability, compatibility with food matrices, sensory quality, and regulatory approval still require systematic evaluation.

## 6. Conclusions

The principal strength of DES synergistic enhancement technologies lies in the combination of designer solvents with targeted process intensification, which provides an efficient and environmentally friendly route for the extraction of natural bioactive compounds. Their core advantage is that DES formulations can be customized according to the nature of the target compound and combined with physical or enzymatic methods to overcome mass transfer limitations. This combination increases extraction efficiency and selectivity without compromising environmental friendliness. These technologies have demonstrated remarkable efficacy in extracting a wide range of bioactive compounds, including polyphenols, alkaloids, terpenoids, and polysaccharides. Further studies should go beyond “phenomenon description” and “condition optimization” toward “mechanism exploration” and “engineering applications.” First, molecular dynamics simulations and in situ characterization methods (e.g., infrared spectroscopy) are needed to clarify the structure–property relationships of DESs and the mechanisms underlying multi-technology synergy. Second, the engineering, economic, and safety issues of industrial scale-up must be addressed urgently, which requires strengthened interdisciplinary cooperation (e.g., chemistry, engineering, and medicine). Together, these efforts will help develop DES synergistic technologies into mature and productive tools that support the green production of natural products.

## Figures and Tables

**Table 1:** Representative yields of bioactive compounds extracted using DES-based synergistic technologies reported in the recent literature.

Source	Bioactive Ingredients	DES Component	Extraction Method	Yield	Reference
*Ficus carica* L.	CMA, PAG, RT, PSL, BET	Gly:Xyl:Fru (3:3:3)	Microwave-assisted extraction	CMA 6.48 mg/g, PAG 16.34 mg/g, RT 5.21 mg/g, PSL 15.22 mg/g, BET 2.48 mg/g	[48]
Spent coffee grounds	Polyphenols	ChCl:1,2-propanediol (1:2)	-	1.4%	[49]
Sour cherry pomace	Polyphenols	ChCl:MalA (1:1)	Microwave- and ultrasound-assisted extraction	3238.3 μg/g	[50]
*Punica granatum* L.	Polyphenols	MalA:Fru:Gly (1:1:1)	Infrared-assisted extraction	152 mg GAE/g DM	[51]
Apple pomace	Polyphenols	betaine:urea (1:1)	-	4.83 mg GAE/g DW	[52]
Cocoa bean shell	Polyphenols	ChCl:lactic acid (1:2)	-	14.44 ± 0.31 mg GAE/g DM	[53]
Olive leaves	Polyphenols	Glycerol:Lysine (3:1)	-	188.39 ± 0.37 mg GAE/g DM	[54]
*Chelidonium majus* L.	Alkaloids	Menthol:Thymol (5:5)	Ultrasound-assisted extraction	12.01 mg GAE/g DM	[17]
*Camptotheca acuminata* Decne dried root powder	Alkaloids	ChCl:FA (1:2)	Ultrasound-assisted extraction	6.08 mg GAE/g DM	[55]
*Zanthoxylum nitidum* (Roxb.) DC roots	Alkaloid	Urea:Propanoic acid (1:2)	Ultrasound–enzyme-assisted extraction	34.56 ± 1.09 mg/g	[56]
Rosemary leaves	Terpenoids	Geraniol:1-dodecanol (1:1)	Ultrasound–enzyme-assisted extraction	5.61 ± 1.96 mg/g	[57]
*Abelmoschus sagittifolius*	Terpenoids	Lactic acid:glucose (2:1)	Ultrasound–microwave-assisted extraction (UMAE)	111 ± 3 mg UA/g dw	[58]
*Angelica sinensis* Radix powder	Essential oils	ChCl:citric acid (1:3)	Microwave-assisted hydrodistillation	1.39%	[59]
*Nardostachys jatamansi*	Essential oils	ChCl:maleic acid (2:1)	Hydrodistillation	1.77%	[60]
*Morchella importuna*	Polysaccharides	ChCl:OA (2:1)	Ultrasound-assisted extraction	5.93 ± 0.07%	[61]
*Sargassum horneri*	Polysaccharides	ChCl:1,2-propanediol (1:2)	Ultrasound-assisted extraction	11.31%	[62]
*Bletilla striata*	Polysaccharides	ChCl:Urea (1:2)	-	55.89%	[63]
*Dioscorea opposita* Thunb	Polysaccharides	ChCl:1,4-butanediol (4:1)	Ultrasound-assisted extraction	15.98 ± 0.15%	[64]
*Ganoderma lucidum*	Polysaccharides	ChCl:Gua:Lac (1:1:1)	-	9.47%	[65]
*Camellia sinensis*	Polysaccharides	ChCl:EG (1:3)	Ultrasound-assisted extraction	15.89 ± 0.13%	[66]
Radix Bupleuri	Polysaccharides	ChCl:Urea (1:3)	Ultrasound–enzyme synergistic extraction	10.10%	[67]

Note: The yields are obtained under different experimental conditions (temperature, time, solid-to-solvent ratio, raw material pretreatment, and analytical methods) and are expressed in varying units depending on the original study. Therefore, these values are intended to illustrate the diversity of DES applications rather than to enable direct quantitative comparison across studies. CMA: caffeoylmalic acid; PAG: psoralic acid-glucoside; RT: quercetin 3-O-rutinoside; PSL: psoralen; BET: bergapten; Gly: glycerol; Xyl: xylitol; Fru: D-(−)-fructose; MalA: L-malic acid; FA: formic acid; ChCl: choline chloride; OA: oxalic acid; Lac: lactic acid; Gua: guaiacol; EG: ethylene glycol.

## Data Availability

No new data were created or analyzed in this study. Data sharing is not applicable to this article.
